# A Comparison of the Nephrotoxicity of Low Doses of Cadmium and Lead

**DOI:** 10.3390/toxics8010018

**Published:** 2020-03-02

**Authors:** Soisungwan Satarug, Glenda C. Gobe, Pailin Ujjin, David A. Vesey

**Affiliations:** 1 National Research Centre for Environmental Toxicology, The University of Queensland, Coopers Plains, Brisbane 4108, Australia; sj.satarug@yahoo.com.au (S.S.); upailin@gmail.com (P.U.); 2 Kidney Disease Research Collaborative, The University of Queensland Faculty of Medicine and Translational Research Institute, Woolloongabba, Brisbane 4102, Australia; g.gobe@uq.edu.au; 3 School of Biomedical Sciences, The University of Queensland, Brisbane 4072, Australia; 4 NHMRC Centre of Research Excellence for CKD.QLD, UQ Health Sciences, Royal Brisbane and Women’s Hospital, Brisbane 4029, Australia; 5 Department of Laboratory Medicine, Chulalongkorn University Faculty of Medicine, Bangkok 10330, Thailand; 6 Department of Nephrology, Princess Alexandra Hospital, Brisbane 4075, Australia

**Keywords:** cadmium, creatinine clearance, creatinine excretion, glomerular filtration rate, lead, nephrotoxicity

## Abstract

Environmental exposure to moderate-to-high levels of cadmium (Cd) and lead (Pb) is associated with nephrotoxicity. In comparison, the health impacts of chronic low-level exposure to Cd and Pb remain controversial. The aim of this study was to therefore evaluate kidney dysfunction associated with chronic low-level exposure to Cd and Pb in a population of residents in Bangkok, Thailand. The mean age and the estimated glomerular filtration rate (eGFR) for 392 participants (195 men and 197 women) were 34.9 years and 104 mL/min/1.73 m^2^, respectively, while the geometric mean concentrations of urinary Cd and Pb were 0.25 μg/L (0.45 μg/g of creatinine) and 0.89 μg/L (1.52 μg/g of creatinine), respectively. In a multivariable regression analysis, the eGFR varied inversely with blood urea nitrogen in both men (β = −0.125, *p* = 0.044) and women (β = −0.170, *p* = 0.008), while inverse associations of the eGFR with urinary Cd (β = −0.132, *p* = 0.043) and urinary Pb (β = −0.130, *p* = 0.044) were seen only in women. An increased urinary level of Cd to the median level of 0.38 μg/L (0.44 μg/g of creatinine) was associated with a decrease in the eGFR by 4.94 mL/min/1.73 m^2^ (*p* = 0.011). The prevalence odds of a reduced eGFR rose 2.5-, 2.9- and 2.3-fold in the urinary Cd quartile 3 (*p* = 0.013), the urinary Cd quartile 4 (*p* = 0.008), and the urinary Pb quartile 4 (*p* = 0.039), respectively. This study suggests that chronic exposure to low-level Cd is associated with a decline in kidney function and that women may be more susceptible than men to nephrotoxicity due to an elevated intake of Cd and Pb.

## 1. Introduction

Cadmium (Cd) and lead (Pb) are environmental toxicants of significant public health concern due to their widespread environmental pollution and persistence, as well as their known adverse impacts on human health, including an enhanced risk of chronic kidney disease (CKD) and various types of cancer [1,2,3,4,5]. The International Agency for Research on Cancer has established Cd as a human carcinogen [6], while the carcinogenicity of chronic Pb exposure in workplace settings has been observed in two large prospective cohort studies [7,8]. Co-exposure to low levels of environmental Cd and Pb has been reported in large population-based studies in the U.S. [9,10,11,12], Canada [13], Taiwan [14], and Korea [15]. About half of the participants in the U.S. National Health and Nutrition Examination Surveys (NHANES) 2007–2012, aged ≥6 years, had blood or urinary levels of Cd and Pb above reported median levels [11].

CKD is the cause of significant human morbidity and mortality. Its high worldwide prevalence and escalating treatment costs make developing strategies to prevent CKD of global importance [16,17,18]. CKD is characterized by albuminuria and/or a decrease of the glomerular filtration rate (GFR) to 60 mL/min/1.73 m^2^ that persists for at least three months [19,20,21]. In theory, the GFR reflects the number of surviving nephrons × the average GFR per nephron [21]. Accordingly, GFR is considered to best indicate nephron function. In practice, the GFR is estimated from equations, including the Chronic Kidney Disease Epidemiology Collaboration (CKD-EPI) equations [19,20,21,22], and is reported as an estimated GFR (eGFR). Prospective cohort studies in Sweden have linked low-level Pb exposure to decreases in the GFR, CKD onset, and end-stage kidney disease [4,23]. In addition, cross-sectional studies have implicated low-level environmental exposure to Cd as a risk factor for CKD in Spain [24], Korea [25], and the U.S. [26,27,28,29].

Elevated dietary Cd intake has been associated with an increased risk of CKD in China [30]. A marked decrease in the GFR has also been observed in residents of an area with Cd pollution in Thailand [2,31,32,33,34,35], as well as in Cd-exposed workers [36,37]. Likewise, marked GFR decreases have been noted in workers and residents of Pb-smelter communities [38]. However, the variable effect of low-level environmental exposure to Cd and Pb on GFR has caused some controversy. Consequently, governments worldwide have not established the necessary regulations to protect their populations. An inverse association has been seen between the GFR and urinary Cd and/or blood Cd [9,28,39,40]. In the opposite direction, other studies have observed a positive association between GFR and urinary Cd [9,12,41,42]. Inverse associations of blood urea nitrogen (BUN) with blood Cd and Pb were noted in a prospective cohort study of premenopausal U.S. women [43]. However, a cross-sectional analysis of data from adolescents (*n* = 2709, aged 12–19 years) who were enrolled in NHANES 2009–2014 reported positive associations of urinary Cd and Pb with an increased eGFR and BUN in models that incorporated urinary creatinine as a covariate [42]. A common practice of normalizing urinary concentrations of Cd to urinary concentrations of creatinine may have caused these disparate findings [33,35,44,45].

The most frequently reported adverse effects of chronic Cd exposure in the general population have included tubular injury and reduced tubular re-absorption, as reflected by elevated urinary N-acetyl-β-D-glucosaminidase (NAG) and β_2_-microglobulin (β_2_MG) levels, respectively [5]. However, despite numerous reports, the observed Cd-linked tubular dysfunction has not been considered to be clinically relevant [5]. Thus, the present study aimed to clarify the impact on kidney function of long-term environmental exposure to low levels of Cd and Pb with a focus on the GFR, a reliable clinical measure of kidney function and diagnosis of CKD. We used the CKD-EPI equations to derive the eGFR and excretion of Cd (E_Cd_) as indicators of body burden. The associations of the eGFR with E_Cd_ and the excretion of Pb (E_Pb_), age, gender, smoking, body iron stores, and BUN (another indicator of kidney effect) were evaluated. For a comparative analysis, E_Cd_ and E_Pb_ were normalized to both the creatinine clearance (C_cr_) and the excretion of creatinine (E_cr_).

## 2. Materials and Methods

### 2.1. Study Population

We assembled archived data from participants who were drawn from residential areas in the Bangkapi suburb of Bangkok, Thailand, between 2001 and 2003. Our Thai urban population project was undertaken based on the global food monitoring system database, which indicated dietary Cd and Pb may exceed the tolerable intake levels 7 µg Cd/kg body weight/week and 25 µg Pb/kg body weight/week in some countries, Thailand included [46]. The Institutional Ethical Committee, Chulalongkorn Medical Faculty Hospital, Chulalongkorn University, Bangkok, Thailand, approved the study protocol (Approval No. 142/2544, 5 October 2001). All participants were apparently healthy and had no history of exposure to Cd or Pb in the workplace. Participants took part in the study after giving informed consent. The health status of participants was assessed by a physical examination and was confirmed by routine urinary and blood chemistry analysis. Smoking, diabetes, hypertension, the regular use of medications, educational level, occupation and family health history were obtained by questionnaires. After the exclusion of participants with incomplete datasets, 392 persons (195 men and 197 women) formed the study cohort.

### 2.2. Specimen Collection and Analysis

Blood samples were collected within 1 h after drinking 300 mL of water following an overnight fast. Urine samples were collected within 3 h of blood sampling. Urine and blood samples were transported on ice to the Department of Laboratory Medicine, Chulalongkorn University Hospital, where plasma samples were prepared for routine chemistry by using an automated system. The assay for plasma and urinary creatinine concentrations was based on the Jaffe reaction, while the plasma ferritin assay was based on an electrochemiluminescence immunoassay (Boehringer Mannheim Elecsys 1010, Roche Diagnostics GmbH, Mannheim, Germany). Aliquots of urine, with 5 mL per aliquot, were shipped on dry ice and kept frozen throughout shipment period. They were delivered to the National Research Centre for Environmental Toxicology, Australia, where they were stored at −80 °C for later analysis. Urinary concentrations of Cd and Pb were determined with inductively-coupled plasma/mass spectrometry (ICP/MS, Agilent 7500, Agilent Technologies, Santa Clara, CA, USA), which had been calibrated with multi-element standards (EM Science, EM Industries, Inc., NJ, USA). Quality assurance and control were conducted with simultaneous analyses of samples of the reference urine Lyphochek^®^ (Bio-Rad, Gladesville, New South Wales, Australia), which contained low- and high-range Cd and Pb levels. A coefficient of variation value of 2.5% was obtained for Cd and Pb in the reference urine. The low limit of detection (LOD) was 0.05 µg/L for urinary Cd and 0.03 µg/L for urinary Pb. The urine samples containing Cd and Pb levels below the LOD were assigned as the LOD divided by the square root of 2. Fifty-eight subjects (14.8%) had urinary Cd levels below the LOD, while 26 subjects (6.6%) had urinary Pb levels below the LOD.

### 2.3. Estimation of Excretion Rates

The procedures for the simultaneous collection of blood and urine samples enabled the normalization of the excretion rates of metals to creatinine clearance (C_cr_) by using the following equation: E_x_/C_cr_ = [x]_u_[cr]_p_/[cr]_u_, where E_x_/C_cr_  =  excretion of x per volume of filtrate; [x]_u_  =  urine concentration of x (mass/volume); [cr]_p_  =  plasma creatinine concentration (mg/dL); and [cr]_u_  =  urine creatinine concentration (mg/dL) [33,35,47]. The normalization of E_x_ to C_cr_ circumvents the effect of muscle mass on E_x_/E_cr_ and [x]_u_/[cr]_u_ while nullifying urine volume (V_u_) as a confounder on concentration ([x]_u_).

As is typical, the excretion of Cd (E_Cd_) was normalized to the excretion of creatinine (E_cr_) as [Cd]_u_/[cr]_u_, where [Cd]_u_  =  urine concentration of Cd (μg/L), and [cr]_u_  =  urine creatinine concentration (mg/dL). The ratio [Cd]_u_/[cr]_u_ was expressed as μg/g of creatinine. This allows for the correction of the urine flow rate (V_u_) on concentration ([x]_u_). However, it introduces another confounder or bias given that E_cr_ is affected by muscle mass and many other factors unrelated to nephron function [48].

### 2.4. Estimated Glomerular Filtration Rates

The eGFR was calculated by using the CKD-EPI equations [19,21]. The male eGFR = 141 × [serum creatinine / 0.9]^Y^ × 0.993^age^, where Y = −0.411 if serum creatinine ≤ 0.9 mg/dL, Y = −1.209 if serum creatinine > 0.9 mg/dL. The female eGFR = 144 × [serum creatinine / 0.7]^Y^ × 0.993^age^, where Y = −0.329 if serum creatinine ≤ 0.7 mg/dL, Y = −1.209 if serum creatinine > 0.7 mg/dL. CKD is defined as an eGFR <60 mL/min/1.73 m^2^ for three months or more [19,22]. CKD stages 1, 2, 3, 4, and 5 corresponded to an eGFR of 90–119, 60–89, 30–59, 15–29, and <15 mL/min/1.73 m^2^, respectively [19,21].

### 2.5. Statistical Analysis

Data were analyzed with SPSS 17.0 (SPSS Inc., Chicago, IL, USA, 2008). The Mann–Whitney U test was used to compare the mean differences between men and women, while the Pearson chi-squared test was used to compare the percentage differences between men and women. The one-sample Kolmogorov–Smirnov test was used to examine departures from a normal distribution of continuous variables, and a base-10 logarithmic transformation was applied to the variables that showed rightward skewing. A multivariable regression model analysis was used to evaluate the association of the eGFR with independent variables including the excretion of Cd and Pb. For each regression model, the coefficient of determination (*R*^2^) value was obtained together with standardized β. A generalized linear model analysis was used to estimate the mean eGFR with adjustment for age, covariates and interactions. Logistic regression analysis was used to estimate the prevalence odds ratio (POR) for the reduced eGFR across the quartiles of excretion of Cd and Pb. The *p*-values ≤0.05 for two-sided tests were assumed to indicate statistical significance.

## 3. Results

### 3.1. Descriptive Characteristics of Study Population

The demographic data, including the blood and urinary biochemistry and other clinical features for the study population of 392 Thai subjects, are shown in Table 1. The overall mean age was 34.9 years. Men were on average 4.1 years younger than women (*p* < 0.001). The mean urinary concentrations of Cd and Pb were 0.25 μg/L (0.45 μg/g of creatinine) and 0.89 μg/L (1.52 μg/g of creatinine), respectively. The mean eGFR (range) was 105 (70−139) mL/min/1.73 m^2^. The percentage of the eGFR <90mL/min/1.73 m^2^ was similar in men and women (12.3% vs. 13.7%, respectively). The percentage of woman with low iron stores (ferritin levels ≤30 µg/L) were six times that of men (22.3% vs 3.6%, *p* < 0.001). Half of the men (49.7%) smoked (8.9 cigarettes per day), with an average duration of 10 years. There was no record of smoking in any of the women.

The mean BUN, serum creatinine, and urinary creatinine were higher in men than women with *p* values of less than 0.001. The mean plasma protein concentration in men and women was similar (*p* = 0.385), as was the mean urinary protein concentration (*p* = 0.162). E_Cd_/E_cr_ and E_Pb_/E_cr_ showed marked differences between men and women. The mean E_Cd_/E_cr_ was 1.3-fold lower in men than women (0.39 vs. 0.51 μg/g of creatinine, *p* < 0.001), while the mean E_Pb_/E_cr_ was 1.9-fold lower in men than women (1.10 vs. 2.10 μg/g of creatinine, *p* < 0.001). Notably, E_Cd_/C_cr_ and E_Pb_/C_cr_ showed little gender differences. The mean for E_Cd_/C_cr_ × 100 in men (0.36 μg/L) was nearly identical to that of women (0.34 μg/L), while the mean E_Pb_/C_cr_ × 100 in men of showed a tendency to be lower than in women (1.02 vs. 1.48 μg/L, *p* = 0.062).

### 3.2. Predictors of eGFR

In the multivariable regression analysis for the eGFR with metal excretion rates normalized to C_cr_ (Table 2), the independent variables (urinary Cd, urinary Pb, age, BUN, serum ferritin, gender and smoking) accounted for 27.2%, 33.4%, 25.9%, 25.4% and 40.2% of the eGFR variability in the entire group, men, women, non-smokers, and smokers, respectively, with the *p* value being <0.001 for the entire group and all subgroups. In the entire group, the eGFR was not associated with urinary Pb (*p* = 0.115), but it showed an inverse association with age (β = −0.436, *p* < 0.001), BUN (β = −0.157, *p* = 0.001) and urinary Cd (β = −0.126, *p* = 0.006).

In a subgroup analysis, the eGFR was inversely associated with urinary Cd (β = −0.132, *p* = 0.043) and urinary Pb (β = −0.130, *p* = 0.044), only in women. In contrast, the eGFR was not associated with urinary Cd (*p* = 0.219) or with urinary Pb (*p* = 0.333) in men, but it showed a positive association with plasma ferritin (β = 0.147, *p* = 0.017). Inverse associations of the eGFR with age and BUN were evident in all subgroups. The strength of an association between the eGFR and BUN was relatively stronger in male smokers (β −0.207, *p* = 0.016), compared with other subgroups, with β values being −0.125 in men (*p* = 0.044), −0.170 in women (*p* = 0.008), and −0.135 in non-smokers (*p* = 0.012).

In an equivalent multivariable regression analysis of the eGFR with metal excretion rates that were normalized to the excretion of creatinine (Table S1), an association between the eGFR and urinary Cd was not evident in the entire group or in any subgroups, as was the association of the eGFR and urinary Pb.

### 3.3. Quantitation of Effects of Cadmium and Lead on the Decline of eGFR

**Figure 1:**
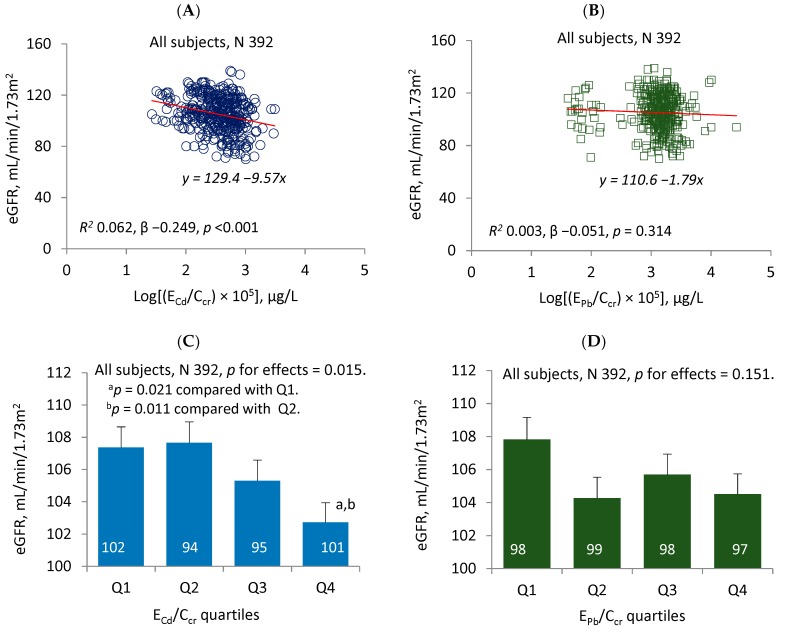
Comparing effects of cadmium and lead on eGFR change. The scatterplots show the relationship between the eGFR and log [excretion of Cd (E_Cd_)/creatinine clearance (C_cr_)) × 10^5^] and between the eGFR and log [excretion of Pb (E_Pb_)/C_cr_) × 10^5^] in all subjects (**A**,**B**). The linear equations and coefficients of determination (*R*^2^) are provided together with standardized β and *p*-values. The bars represent the mean values for the eGFR across urinary Cd and urinary Pb quartiles (**C**,**D**) with adjustments for various covariates and potential interactions. The numbers of subjects are provided for all subgroups. The geometric mean (GM) values (standard deviation) for E_Cd_/C_cr_ × 100 in urinary Cd quartiles 1, 2, 3 and 4 were 0.12 (0.05), 0.30 (0.05), 0.48 (0.07) and 0.88 (0.44) µg/L, respectively. The GM (SD) for E_Pb_/C_cr_ × 100 in urinary Pb quartiles 1, 2, 3 and 4 are 0.41 (0.36), 1.26 (0.10), 1.63 (0.14) and 2.73 (2.86) µg/L, respectively.

Figure 1 provides the results of a quantitative analysis of changes in the eGFR that was done by using metal excretion rates that were normalized to creatinine clearance. In the scatterplot of the eGFR against E_Cd_/C_cr_, a moderate inverse association was evident (β −0.249, *p* < 0.001) (Figure 1A). Six point two % of the eGFR reduction (*R^2^* = 0.062) could be attributed to Cd. An inverse association was evident also from the scatterplot of the eGFR against E_Pb_/C_cr_ (Figure 1B). However, the strength of eGFR-E_Pb_/C_cr_ association was insignificant (*p* = 0.314), and as little as 0.3% of eGFR variation could be attributed to Pb.

A generalized linear model (GLM) was then used to estimate the mean eGFR values for subgroups stratified by the quartiles of E_Cd_/C_cr_ (Figure 1C) and the quartiles of E_Pb_/C_cr_ (Figure 1D). After adjustments for age, covariates and the interactions, a negative effect of Cd on the eGFR was evident (*p* = 0.015). The estimated mean eGFR (standard error of mean, SEM) for males and females with urinary Cd in the fourth quartile was, respectively, 4.65 (1.72) and 4.94 (1.70) mL/min/1.73 m^2^ lower than those with urine Cd in the first quartile (*p* = 0.021) and with urine Cd in the second quartile (*p* = 0.011), respectively. Distinct from Cd, the relationship between Pb and the eGFR was negligible and insignificant (*p* = 0.151) (Figure 1D).

Figure S1 provides the results of an equivalent quantitative analysis of changes in the eGFR that was done by using metal excretion rates that were normalized to the excretion of creatinine. In the scatterplot of the eGFR against E_Cd_/E_cr_ (Figure S1A), an inverse association between the eGFR and E_Cd_/E_cr_ was evident (β = −0.104, *p* = 0.040), but this relationship was weakened and became insignificant (*p* = 0.763) after adjustments for age, covariates and interactions (Figure S1C). In contrast, the scatterplot of the eGFR against E_Pb_/E_cr_ indicated a marginal but non-significant positive association between the eGFR and E_Pb_/E_cr_ (β = 0.080, *p* = 0.115) (Figure S1B). After adjustments for age, covariates and interactions, there were significant increases in eGFR levels across E_Pb_/E_cr_ quartiles (*p* = 0.010) (Figure S1D). The estimated mean eGFR (SEM) for subjects with E_Pb_/E_cr_ in the fourth quartile was 5.89 (1.77) mL/min/1.73 m^2^ higher than those with E_Pb_/E_cr_ in the second quartile (*p* = 0.003).

### 3.4. The Prevalence Odds of Reduced eGFR across the Quartiles of Urinary Cd and Urinary Pb

Table 3 provides the results of a logistic regression analysis of the POR for the reduced eGFR, defined as the eGFR at the 25^th^ percentile or below (≤96 mL/min/1.73 m^2^). The POR for the reduced eGFR showed an inverse association with age (β= −0.071, *p* < 0.001) and gender (β = −1.020, *p* = 0.003). In addition, the POR for the reduced eGFR appeared to rise with urinary Cd in a dose-dependent manner. The POR for the reduced eGFR was 2.87 (95% CI: 1.32, 6.24), 2.51 (95% CI: 1.22, 5.18) and 1.70 (95% CI: 0.875, 3.29) in the urinary Cd quartile 4 (*p* = 0.008), quartile 3 (*p* =0.013) and quartile 1 (*p* = 0.117), respectively. The POR for the reduced eGFR was not associated with the urinary Pb quartile 2 (*p* = 0.198) or quartile 3 (*p* = 0.744), but it rose to 2.23 (95% CI: 1.04, 4.78) in the urinary Pb quartile 4 (*p* = 0.039).

Table S2 provides the results of an equivalent logistic regression analysis for the reduced eGFR that was done by using urinary Cd and urinary Pb that were normalized to the excretion of creatinine. In this analysis, the POR for the reduced eGFR only showed an inverse association with age (β = −0.080, *p* < 0.001). No associations were seen between the reduced eGFR and urinary Cd or Pb in any quartiles of urinary Cd or Pb.

## 4. Discussion

None of the participants in the present study had been exposed to metals in the workplace, and their urinary concentrations of Cd and Pb were thus presumed to reflect environmental sources, notably diet [5,49]. The urinary Cd and Pb concentrations were similar to the data obtained from population-based studies in the U.S. [9,10,11,12], Canada [13], Taiwan [14] and Korea [15]. The eGFR was calculated by using CKD-EPI equations, which are considered to be the most accurate equations for the eGFR [20,22]. The CKD-EPI equations have been validated by using inulin clearance [22]. The overall mean eGFR for participants was 105 mL/min/1.73 m^2^, ranging between 70 and 139 mL/min/1.73 m^2^. The wide range of variation in the eGFR was congruent with the notion that a normal level of the GFR could vary widely [20]. Both physiological and pathological conditions are known to affect the GFR, and the mean GFR in young adult Caucasians is approximately 125 mL/min/1.73 m^2^ [20].

In a regression model in which all subjects were included, the eGFR levels varied inversely with age, BUN and E_Cd_/C_cr_, but they did not vary with E_Pb_/C_cr_ (Table 2). The inverse association between the eGFR and BUN was expected, as urea is one of the metabolic waste products that is eliminated through kidneys. Of note, however, while the inverse associations of the eGFR with age and BUN were present in all subgroups, the association between the eGFR and E_Cd_/C_cr_ reached statistical significance levels in non-smokers (men and women). In addition, the eGFR levels in women varied inversely with both E_Cd_/C_cr_ (β= −0.170, *p* = 0.008) and E_Pb_/C_cr_ (β= −0.132, *p* = 0.043). These data could be interpreted to suggest that levels of Cd and Pb intake from the diet were sufficient to produce adverse effects on nephrons and subsequently reduce the elimination rate of urea. They also suggested that women may be more susceptible than men to renal effects of elevated Cd and Pb intake levels. Supporting this argument is the fact that blood Cd and Pb both were associated with BUN in a prospective study in the U.S. that included 259 premenopausal women, where one-third of them had eGFR levels <90 mL/min/1.73 m^2^ and stage 1 CKD [43]. In addition, urinary Cd and Pb were associated with increased BUN in adolescents enrolled in NHANES 2009–2014 [42].

In a multivariable regression analysis that was done by using urinary Cd and Pb that were normalized to the excretion of creatinine (Table S1), the eGFR did not show significant relationships with E_Cd_/E_cr_ or E_Pb_/E_cr_ in any subgroups. Normalizing to the excretion of creatinine was done to correct for urine dilution. This practice, however, inevitably introduces confounders and often creates gender bias. In general, men have a higher muscle mass than women, and, consequently, the mean E_Cd_/E_cr_ was lower in men than women (Table 1). In effect, the health risks associated with Cd exposure in men could have been underestimated. The mean E_Cd_/C_cr_ in men was almost identical to that of women (Table 1). It is increasingly recognized that creatinine adjustment is problematic, and urine specific gravity has been used to correct for dilution effects [9,44,45,50,51,52]. Herein, we have demonstrated the utility of normalizing excretion rate of metals to creatinine clearance that only required simultaneous urine and blood sampling together with the equations, given in Section 2.3 [33,35].

In a quantitative analysis for an effect of Pb, a non-significant association between the eGFR and E_Pb_/C_cr_ was evident (Figure 1D). Furthermore, the POR for the reduced eGFR did not increase with E_Pb_/C_cr_ in a dose-dependent manner (Table 3). Though urinary Pb is not a good indicator of Pb body burden, it does not rule out the possibility for a glomerular effect of Pb. Blood Pb levels ≥2.4 μg/dL were associated with a 1.56-fold increase in risk of eGFR levels of <60 mL/min/1.73 m^2^ in adults who enrolled in NHANES 1999–2006 [26]. However, the absence of a dose–response relationship between the E_Pb_/C_cr_ quartiles and POR for the reduced eGFR may suggest that the levels of environmental exposure to Pb that were experienced by participants in this study were below a nephrotoxicity threshold limit for Pb.

In a quantitative analysis for an effect of Cd (Figure 1C), an increment of E_Cd_/C_cr_ to the median level (0.38 μg/L) was associated with a significant decrease in the eGFR (an approximate of 5 mL/min/1.73 m^2^). An increment of E_Cd_/C_cr_ to the 75^th^ percentile level (0.62 μg/L) was not associated with a further decrease in the eGFR level. In the logistic regression analysis (Table 3), the POR for the reduced eGFR rose by 2.51 and 2.87 fold as the E_Cd_/C_cr_ rose to the median level and the 75^th^ percentile, respectively. The median E_Cd_/C_cr_ level could thus be considered to represent the lowest urinary Cd level that was associated with observed adverse effect among the participants in the present study. The median and 75^th^ percentile levels of E_Cd_/Ecr corresponded to 0.44 and 0.76 μg/g of creatinine, respectively.

Of relevance, urinary Cd level of 0.8 μg/g of creatinine has been found to be associated with a significant eGFR decrease in Swedish women, aged 53–64 years [39]. In addition, U.S. population studies (NHANES) have provided a rich data source that links Cd and Pb exposure indicators to increased risk of CKD. In NHANES 1999–2006, urinary Cd levels ≥1µg/L and blood Cd levels ≥0.6 μg/L were associated, respectively, with 1.48- and 1.32-fold increases in the risk of a low eGFR, defined as an eGFR <60 mL/min/1.73 m^2^ [26,27]. In NHANES 2011–2012, blood Cd levels >0.53 μg/L were associated with 2.21-fold increases in risk of low eGFR [53].

In NHANES 2007–2012, blood Cd levels >0.61 μg/L were associated with a 1.80-fold increase in the risk of a low GFR [28]. In addition, the mean eGFR in women with hypertension and blood Cd in the highest quartile was 5.77 mL/min/1.73 m^2^ lower than that of normotensive women who had blood Cd in the lowest quartile [28]. Of interest, an additional effect of hypertension on Cd-related GFR reduction has been seen in residents of an area of Thailand with Cd pollution: Those with hypertension, on average, had a 4.6 mL/min/1.73 m^2^ lower eGFR compared with the mean eGFR of normotensive subjects who had similarly high urinary Cd levels [33].

In a Swedish women study, urinary Cd levels associated with a glomerular effect (eGFR decline) of 0.8 µg/g of creatinine were close to urinary Cd levels of 0.67 µg/g of creatinine that were associated with tubular injury based on urinary NAG levels [39]. These data challenge a long-held view that tubular effects occur long before the glomerular effect becomes apparent. A recent quantitative analysis of excreted Cd in relation to levels of the eGFR, urinary NAG, and β_2_MG suggested a decrease in the GFR to be an early effect, given that excreted Cd emanates from injured tubular cells and that the injury leads to nephron atrophy, a decreased eGFR, and impaired reabsorption of filtered β_2_MG [35]. Accordingly, it can be hypothesized that sufficient tubular injury induced by Cd disables glomerular filtration, destroys nephrons, and causes glomerulosclerosis, interstitial inflammation, fibrosis and CKD [35,54].

A lack of association between the eGFR and urinary Cd was reported in a cross-sectional study in Japan [55] in which eGFR values were derived for 1200 women by using a serum creatinine-based eGFR estimating equation for Japanese women. In this Japanese study, 222 women who were aged 42–79 years (mean 61.9) were drawn from a control area without Cd pollution, based on the Cd content in rice, which is a dietary staple, while 636 and 355 women of the similar age range were drawn from two areas with Cd pollution. Though the Cd intake levels from rice in the two Cd pollution areas were higher than those of the control group, the mean eGFR values in these three areas were similar. In addition, the eGFR levels were unrelated to urinary Cd, but they were related to age. These data suggested that the eGFR was not associated with Cd body burden in the Japanese population with relatively high levels of Cd intake [55]. A longitudinal study is required to dispute or confirm the observation made in this Japanese study. It is noteworthy, however, that a prospective study in a Cd pollution area in Thailand reported a progressive decrease in the eGFR over a five-year observation period [31].

Distinct from the Japanese study, an inverse association between the eGFR and urinary Cd was observed in the present study. The study Thai women were younger (the mean age of 36.9) and had lower urinary Cd levels (mean urinary of 0.51 µg/g of creatinine) compared to the Japanese women in the control area (mean age of 61.9) and mean urinary Cd of 3.03 µg/g of creatinine. The mean eGFR (range) was 106 (72−139) and 79.8 (30.6−130) mL/min/1.73 m^2^ in the Thai and Japanese studies, respectively. The eGFR-urinary Cd association in the Thai study could have been due to younger age and lower Cd intake levels compared with the Japanese study. In addition, it is conceivable that the functional expressions of Cd-induced nephrotoxicity in low-dose and high-dose exposure conditions are different. For instance, a low-level environmental exposure to Cd has been implicated in the pathogenesis of hypertension, a known cause and consequence of CKD, in a longitudinal study in the U.S. [56], while blood Cd as low as 0.4 µg/L was associated, respectively, with 1.54- and 2.38-fold increases in risk of hypertension in Caucasian women and Mexican-American women who were aged ≥20 years and enrolled in NHANES 1999–2006 [57]. In stark contrast, a chronic high-dose Cd exposure has not been found to be associated with hypertension in Japanese population studies [58,59].

In the present study, chronic low-level exposure to Cd, indicated by a urinary Cd level as low as 0.44 μg/g of creatinine was associated with a decrease in the eGFR by 5 mL/min/1.73m^2^. This low urinary Cd level was also associated with a 2.5 fold increase in the prevalence odds of eGFR levels <96 mL/min/1.73 m^2^.These findings support the large number of population-based studies that have suggested that low-environmental exposure to Cd may increase the risk of CKD, thereby raising the possibility for a role of Cd exposure in current epidemics of CKD. It is noteworthy that the reported toxic urinary Cd levels did not exceed the toxicity threshold limit of 5.24 μg/g of creatinine that was established by the Food and Agriculture Organization of the United Nations (FAO) World Health Organization (WHO) [49,60]. We suggest that the current urinary Cd threshold limit does not afford health protection and should be lowered.

A small observable decrease in the eGFR that is attributable to long-term environmental exposure to Cd was expected, as the participants in our study were relatively young, with an overall mean age of 34.9 years. However, because dietary exposure to Cd and Pb is inevitable for most people, exposure to these toxic metals is likely to continue, leading to a further reduction in the GFR. In addition, the GFR may continue to decrease presumably due to mobilization of liver Cd and bone Pb to kidneys [5]. Even a small increase in the risk of CKD can result in many affected people, given that environmental exposure to Cd and Pb is widespread.

In conclusion, our analysis of archived data provides evidence that links environmental exposure to Cd to GFR decline, even when dietary intake levels of Cd are low. This glomerular effect of low-level environmental exposure to Cd was demonstrable only when urinary Cd concentrations that were normalized to creatinine clearance. Women appeared to be more susceptible than men to toxicity due to an elevated dietary intake of Cd and Pb.

## 5. Strengths and Limitations

The strengths of this study include the community-based recruitment of apparently healthy women and men who were relatively young, as well as the fact that simultaneous blood and urine sampling was undertaken at the same time of the day, thereby reducing diurnal variation of the GFR. The normalization of excretion rates of Cd and Pb to creatinine clearance was an additional strength because confounding effects of muscle mass and urine flow rate were both eliminated. Furthermore, environmental sources of Cd and Pb were relatively homogenous, as none of the participants had occupational exposure to metals. The limitations of this study were archived data with no availability to access the same people for long term comparisons, a modest sample size, and a cross-sectional design, which limited a causal inference of Cd and Pb exposure on the observed GFR reduction.

## Figures and Tables

**Table 1:** Descriptive characteristics of study population.

Parameters/Factors	All Subjects *n* = 392	Men *n* = 195	Women *n* = 197	*p*-Values
Age, years	34.9 ± 9.6 (16−60)	32.8 ± 8.8 (16−57)	36.9 ± 10 (19−60)	<0.001 *
Smoking (%)	24.7	49.7	0	<0.001 *
Serum ferritin, μg/L	89.0 ± 134 (3−378)	159 ± 153 (14−978)	50.0 ± 61.3 (3−353)	<0.001 *
Low body iron stores (%) ^a^	13.0	3.6	22.3	<0.001 *
eGFR, mL/min/1.73 m^2 b^	105 ± 14 (70−139)	104 ± 14 (70−138)	106 ± 13 (72−139)	0.170
eGFR <90 mL/min/1.73 m^2^	13.0	12.3	13.7	0.681
BUN, mg/dL	11.1 ± 2.8 (5−24)	11.8 ± 2.8 (5−24)	10.4 ± 2.8 (5−19)	<0.001 *
Total plasma protein, g/dL	7.85 ± 0.46 (6−9.1)	7.86 ± 0.46 (6−9.1)	7.84 ± 0.47 (6.9−9.1)	0.385
Plasma creatinine, mg/dL	0.82 ± 0.16 (0.5−1.3)	0.95 ± 0.11 (0.5−1.3)	0.71 ± 0.10 (0.5−1.0)	<0.001 *
Urine creatinine, mg/dL	59.5 ± 68.4 (7.2−377)	74.2 ± 79.1 (11−377)	47.8 ± 49.1 (7.2−294)	<0.001 *
Total urine protein, mg/dL	1.60 ± 8.41 (0.02−70)	1.80 ± 9.14 (0.02−65)	1.43 ± 7.57 (0.02−75)	0.162
Urinary concentrations				
Cd, μg/L	0.25 ± 0.68 (0.04−9.4)	0.28 ± 0.84 (0.04−9.4)	0.23 ± 0.49 (0.04−4.2)	0.117
Pb, μg/L	0.89 ± 1.73 (0.02−19)	0.80 ± 1.66 (0.02−13)	1.00 ± 1.80 (0.1−19)	0.239
Urinary metals normalized to excretion of creatinine				
E_Cd_/E_cr_, µg/g of creatinine	0.45 ± 0.46 (0.03−3.8)	0.39 ± 0.46 (0.03−3.8)	0.51 ± 0.46 (0.04−2.4)	<0.001 *
E_Pb_/E_cr_, µg/g of creatinine	1.52 ± 2.16 (0.05−33)	1.10 ± 1.42 (0.05−13)	2.10 ± 2.62 (0.05−33)	<0.001 *
Urinary metals normalized to creatinine clearance				
E_Cd_/C_cr_ × 100, μg/L	0.35 ± 0.38 (0.03−3.1)	0.36 ± 0.42 (0.03−3.1)	0.34 ± 0.34 (0.03−1.9)	0.842
E_Pb_/C_cr_ × 100, μg/L	1.23 ± 1.70 (0.04−27)	1.02 ± 1.20 (0.04−19)	1.48 ± 2.08 (0.04−27)	0.062

^a^ Low body iron stores were defined as plasma ferritin ≤30 μg/L. ^b^ eGFR = estimated glomerular filtration rate, determined with Chronic Kidney Disease Epidemiology Collaboration (CKD−EPI) equations [19,21]. Data for age and the eGFR are arithmetic mean values ± standard deviation (SD). Data for all other continuous variables are geometric mean ± SD values. Numbers in parentheses are range. * *p* ≤ 0.05 indicate mean or % differences between men and women based on the Mann–Whitney U-test or the Pearson chi-squared test, respectively.

**Table 2:** Predictors of the estimated glomerular filtration rate (eGFR).

Independent Variables	eGFR, mL/min/1.73 m^2^									
	All, *n* = 392		Men, *n* = 195		Women, *n* = 197		Non-Smokers, *n* = 295		Smokers, *n* = 97	
	β	*p*	β	*p*	β	*p*	β	*p*	β	*p*
Age	−0.436	<0.001 *	−0.501	<0.001 *	−0.378	<0.001 *	−0.405	<0.001 *	−0.510	<0.001
BUN	−0.157	0.001 *	−0.125	0.044 *	−0.170	0.008 *	−0.135	0.012 *	−0.207	0.016 *
Urine Cd	−0.126	0.006 *	−0.082	0.219	−0.132	0.043 *	−0.119	0.026 *	−0.122	0.182
Urine Pb	−0.072	0.115	−0.060	0.333	−0.130	0.044	−0.092	0.092	−0.056	0.513
Ferritin	0.076	0.156	0.147	0.017 *	0.002	0.969	0.059	0.330	0.101	0.227
Gender	0.222	0.001 *	−	−	−	−	0.208	0.001 *	−	−
Smoking	0.075	0.166	0.062	0.317	−	−	−	−	−	−
Adjusted *R^2^*	0.272	<0.001 ^†^	0.334	<0.001 ^†^	0.259	<0.001 ^†^	0.254	<0.001 ^†^	0.402	<0.001 ^†^

The eGFR is a continuous dependent variable. Independent variables are listed in the first column, including urine Cd as log [(E_Cd_/C_cr_) × 10^5^], μg/L and urine Pb as log [(E_Pb_/C_cr_) × 10^5^]. A standardized regression coefficient β indicates the strength of an association between the eGFR and an independent variable. * *p* ≤ 0.05 identifies statistically significant associations. An adjusted *R^2^* value indicates the fraction of eGFR variation explained by independent variables. ^†^ *p* ≤ 0.05 indicates that the model explained a significant variability of eGFR levels.

**Table 3:** Prevalence odds ratios for the reduced eGFR across the E_Cd_/C_cr_ and E_Pb_/C_cr_ quartiles.

Independent Variables/Factors	eGFR Levels <96 mL/min/1.73 m^2^				
	β Coefficients	POR ^a^	95% CI for POR		*p*
	(SE)		Lower	Upper	Value
Age (years)	−0.071 (0.015)	0.931	0.904	0.959	<0.001
Gender	−1.020 (0.345)	0.361	0.184	0.709	0.003
Smoking	−0.475 (0.367)	0.622	0.303	1.277	0.195
Low body iron store status ^b^	−0.015 (0.437)	0.985	0.418	2.320	0.972
E_Cd_/C_cr_ × 100, μg/L					
Q1 (0.03−0.21)	Referent	1.000	1.000	1.000	
Q2 (0.22−0.38)	0.529 (0.338)	1.697	0.875	3.291	0.117
Q3 (0.39−0.61)	0.920 (0.370)	2.510	1.216	5.181	0.013
Q4 (0.62−3.10)	1.053 (0.396)	2.867	1.319	6.236	0.008
E_Pb_/C_cr_ × 100, μg/L					
Q1 (0.04−1.07)	Referent	1.000	1.000	1.000	
Q2 (1.08−1.42)	0.469 (0.365)	1.598	0.782	3.265	0.198
Q3 (1.43−1.93)	0.115 (0.535)	1.122	0.562	2.241	0.744
Q4 (1.94−26.5)	0.803 (0.388)	2.233	1.043	4.780	0.039

^a^ POR = prevalence odds ratios for eGFR levels ≤96 mL/min/1.73 m^2^. The eGFR 96 mL/min/1.73 m^2^ corresponds to the 25th percentile eGFR. ^b^ Low iron store status was defined as serum ferritin levels ≤ 30μg/L. * *p* ≤ 0.05 indicates a statistically significant increment of POR, compared with the reference. The GM (SD) for E_Cd_/C_cr_ and E_Pb_/C_cr_ together with number of subjects in all urinary Cd and urinary Pb quartiles are as in Figure 1.

## Supplementary Materials

The following are available online at https://www.mdpi.com/2305-6304/8/1/18/s1, Figure S1: Comparing effects of E_Cd_/E_cr_ and E_Pb_/E_cr_ on eGFR change; Table S1: Multivariable regression analysis for association of eGFR with E_Cd_/E_cr_ and E_Pb_/E_cr_; Table S2: Prevalence odds ratios for reduced eGFR across E_Cd_/E_cr_ quartiles and E_Pb_/E_cr_ quartiles.

## References

[1] Satarug: S., Garrett S.H., Sens M.A., Sens D.A. (2010). Cadmium, environmental exposure, and health outcomes. Environ. Health Perspect..

[2] Satarug S., Ruangyuttikarn W., Nishijo M., Ruiz P. (2018). Urinary cadmium threshold to prevent kidney disease development. Toxics.

[3] Shefa S.T., Héroux P. (2017). Both physiology and epidemiology support zero tolerable blood lead levels. Toxicol. Lett..

[4] Harari F., Sallsten G., Christensson A., Petkovic M., Hedblad B., Forsgard N., Melander O., Nilsson P.M., Borné Y., Engström G. (2018). Blood lead levels and decreased kidney function in a population-based cohort. Am. J. Kidney Dis..

[5] Satarug S. (2018). Dietary cadmium intake and its effects on kidneys. Toxics.

[6] IARC (International Agency for Research on Cancer) (1993). Cadmium and cadmium compounds. Beryllium, Cadmium, Mercury and Exposures in the Glass Manufacturing Industry.

[7] Liao L.M., Friesen M.C., Xiang Y.B., Cai H., Koh D.H., Ji B.T., Yang G., Li H.L., Locke S.J., Rothman N. (2016). Occupational lead exposure and associations with selected cancers: The Shanghai men’s and women’s health study cohorts. Environ. Health Perspect..

[8] Steenland K., Barry V., Anttila A., Sallmén M., McElvenny D., Todd A.C., Straif K. (2017). A cohort mortality study of lead-exposed workers in the USA, Finland and the UK. Occup. Environ. Med..

[9] Buser M.C., Ingber S.Z., Raines N., Fowler D.A., Scinicariello F. (2016). Urinary and blood cadmium and lead and kidney function: NHANES 2007–2012. Int. J. Hyg. Environ. Health.

[10] Wang W., Schaumberg D.A., Park S.K. (2016). Cadmium and lead exposure and risk of cataract surgery in U.S. adults. Int. J. Hyg. Environ. Health.

[11] Shim Y.K., Lewin M.D., Ruiz P., Eichner J.E., Mumtaz M.M. (2017). Prevalence and associated demographic characteristics of exposure to multiple metals and their species in human populations: The United States NHANES, 2007–2012. J. Toxicol. Environ. Health A.

[12] Jin R., Zhu X., Shrubsole M.J., Yu C., Xia Z., Dai Q. (2018). Associations of renal function with urinary excretion of metals: Evidence from NHANES 2003–2012. Environ. Int..

[13] Saravanabhavan G., Werry K., Walker M., Haines D., Malowany M., Khoury C. (2017). Human biomonitoring reference values for metals and trace elements in blood and urine derived from the Canadian Health Measures Survey 2007–2013. Int. J. Hyg. Environ. Health.

[14] Liao K.W., Pan W.H., Liou S.H., Sun C.W., Huang P.C., Wang S.L. (2019). Levels and temporal variations of urinary lead, cadmium, cobalt, and copper exposure in the general population of Taiwan. Environ. Sci. Pollut. Res. Int..

[15] Kim N.S., Ahn J., Lee B.K., Park J., Kim Y. (2017). Environmental exposures to lead, mercury, and cadmium among South Korean teenagers (KNHANES 2010–2013): Body burden and risk factors. Environ. Res..

[16] De Nicola L., Zoccali C. (2016). Chronic kidney disease prevalence in the general population: Heterogeneity and concerns. Nephrol. Dial. Transplant..

[17] Glassock R.J., David G., Warnock D.G., Delanaye P. (2017). The global burden of chronic kidney disease: Estimates, variability and pitfalls. Nat. Rev. Nephrol..

[18] George C., Mogueo A., Okpechi I., Echouffo-Tcheugui J.B., Kengne A.P. (2017). Chronic kidney disease in low-income to middle-income countries: The case for increased screening. BMJ Glob. Health.

[19] Levey A.S., Stevens L.A., Schmid C.H., Zhang Y., Castro A.F., Feldman H.I., Kusek J.W., Eggers P., Van Lente F., Greene T. (2009). A new equation to estimate glomerular filtration rate. Ann. Intern. Med..

[20] Levey A.S., Inker L.A., Coresh J. (2014). GFR estimation: From physiology to public health. Am. J. Kidney Dis..

[21] Levey A.S., Becker C., Inker L.A. (2015). Glomerular filtration rate and albuminuria for detection and staging of acute and chronic kidney disease in adults: A systematic review. JAMA.

[22] White C.A., Allen C.M., Akbari A., Collier C.P., Holland D.C., Day A.G., Knoll G.A. (2019). Comparison of the new and traditional CKD-EPI GFR estimation equations with urinary inulin clearance: A study of equation performance. Clin. Chim. Acta.

[23] Sommar J.N., Svensson M.K., Björ B.M., Elmståhl S.I., Hallmans G., Lundh T., Schön S.M., Skerfving S., Bergdahl I.A. (2013). End-stage renal disease and low level exposure to lead, cadmium and mercury; a population-based, prospective nested case-referent study in Sweden. Environ. Health.

[24] Grau-Perez M., Pichler G., Galan-Chilet I., Briongos-Figuero L.S., Rentero-Garrido P., Lopez-Izquierdo R., Navas-Acien A., Weaver V., García-Barrera T., Gomez-Ariza J.L. (2017). Urine cadmium levels and albuminuria in a general population from Spain: A gene-environment interaction analysis. Environ. Int..

[25] Myong J.P., Kim H.R., Baker D., Choi B. (2012). Blood cadmium and moderate-to-severe glomerular dysfunction in Korean adults: Analysis of KNHANES 2005−2008 data. Int. Arch. Occup. Environ. Health.

[26] Navas-Acien A., Tellez-Plaza M., Guallar E., Muntner P., Silbergeld E., Jaar B., Weaver V. (2009). Blood cadmium and lead and chronic kidney disease in US adults: A joint analysis. Am. J. Epidemiol..

[27] Ferraro P.M., Costanzi S., Naticchia A., Sturniolo A., Gambaro G. (2010). Low level exposure to cadmium increases the risk of chronic kidney disease: Analysis of the NHANES 1999–2006. BMC Public Health.

[28] Madrigal J.M., Ricardo A.C., Persky V., Turyk M. (2018). Associations between blood cadmium concentration and kidney function in the U.S. population: Impact of sex, diabetes and hypertension. Environ. Res..

[29] Zhu X.J., Wang J.J., Mao J.H., Shu Q., Du L.Z. (2019). Relationships between cadmium, lead and mercury levels and albuminuria: Results from the National Health and Nutrition Examination Survey Database 2009−2012. Am. J. Epidemiol..

[30] Shi Z., Taylor A.W., Riley M., Byles J., Liu J., Noakes M. (2017). Association between dietary patterns, cadmium intake and chronic kidney disease among adults. Clin. Nutr..

[31] Swaddiwudhipong W., Limpatanachote P., Mahasakpan P., Krintratun S., Punta B., Funkhiew T. (2012). Progress in cadmium-related health effects in persons with high environmental exposure in northwestern Thailand: A five-year follow-up. Environ. Res..

[32] Swaddiwudhipong W., Nguntra P., Kaewnate Y., Mahasakpan P., Limpatanachote P., Aunjai T., Jeekeeree W., Punta B., Funkhiew T., Phopueng I. (2015). Human health effects from cadmium exposure: Comparison between persons living in cadmium-contaminated and non-contaminated areas in northwestern Thailand. Southeast Asian J. Trop. Med. Public Health.

[33] Satarug S., Boonprasert K., Gobe G.C., Ruenweerayut R., Johnson D.W., Na-Bangchang K., Vesey D.A. (2018). Chronic exposure to cadmium is associated with a marked reduction in glomerular filtration rate. Clin. Kidney J..

[34] Satarug S., Vesey D.A., Nishijo M., Ruangyuttikarn W., Gobe G.C. (2019). The inverse association of glomerular function and urinary β2-MG excretion and its implications for cadmium health risk assessment. Environ. Res..

[35] Satarug S., Vesey D.A., Ruangyuttikarn W., Nishijo M., Gobe G.C., Phelps K.R. (2019). The source and pathophysiologic significance of excreted cadmium. Toxics.

[36] Roels H.A., Lauwerys R.R., Buchet J.P., Bernard A.M., Vos A., Oversteyns M. (1989). Health significance of cadmium induced renal dysfunction: A five year follow up. Occup. Environ. Med..

[37] Jarup L., Persson B., Elinder C.G. (1995). Decreased glomerular filtration rate in solderers exposed to cadmium. Occup. Environ. Med..

[38] Reilly R., Spalding S., Walsh B., Wainer J., Pickens S., Royster M., Villanacci J., Little B.B. (2018). Chronic environmental and occupational lead exposure and kidney function among African Americans: Dallas Lead Project II. Int. J. Environ. Res. Public Health.

[39] Akesson A., Lundh T., Vahter M., Bjellerup P., Lidfeldt J., Nerbrand C., Samsioe G., Strömberg U., Skerfving S. (2005). Tubular and glomerular kidney effects in Swedish women with low environmental cadmium exposure. Environ. Health Perspect..

[40] Hwangbo Y., Weaver V.M., Tellez-Plaza M., Guallar E., Lee B.K., Navas-Acien A. (2011). Blood cadmium and estimated glomerular filtration rate in Korean adults. Environ. Health Perspect..

[41] Weaver V.M., Kim N.S., Jaar B.G., Schwartz B.S., Parsons P.J., Steuerwald A.J., Todd A.C., Simon D., Lee B.K. (2011). Associations of low-level urine cadmium with kidney function in lead workers. Occup. Environ. Med..

[42] Sanders A.P., Mazzella M.J., Malin A.J., Hair G.M., Busgang S.A., Saland J.M., Curtin P. (2019). Combined exposure to lead, cadmium, mercury, and arsenic and kidney health in adolescents age 12–19 in NHANES 2009–2014. Environ. Int..

[43] Pollack A.Z., Mumford S.L., Mendola P., Perkins N.J., Rotman Y., Wactawski-Wende J., Schisterman E.F. (2015). Kidney biomarkers associated with blood lead, mercury, and cadmium in premenopausal women: A prospective cohort study. J. Toxicol. Environ Health A.

[44] Weaver V.M., Vargas G.G., Silbergeld E.K., Rothenberg S.J., Fadrowski J.J., Rubio-Andrade M., Parsons P.J., Steuerwald A.J., Navas-Acien A., Guallar E. (2014). Impact of urine concentration adjustment method on associations between urine metals and estimated glomerular filtration rates (eGFR) in adolescents. Environ. Res..

[45] Weaver V.M., Kotchmar D.J., Fadrowski J.J., Silbergeld E.K. (2016). Challenges for environmental epidemiology research: Are biomarker concentrations altered by kidney function or urine concentration adjustment?. J. Expo. Sci. Environ. Epidemiol..

[46] Galal-Gorchev H. (1993). Dietary intake, levels in food and estimated intake of lead, cadmium, and mercury. Food Addit. Contam..

[47] Phelps K.R., Stote K.S., Mason D. (2014). Tubular calcium reabsorption and other aspects of calcium homeostasis in primary and secondary hyperparathyroidism. Clin. Nephrol..

[48] Heymsfield S.B., Arteaga C., McManus C., Smith J., Moffitt S. (1983). Measurement of muscle mass in humans: Validity of the 24-h urinary creatinine method. Am. J. Clin. Nutr..

[49] Satarug S., Vesey D.A., Gobe G.C. (2017). Health risk assessment of dietary cadmium intake: Do current guidelines indicate how much is safe?. Environ. Health Perspect..

[50] Jenny-Burri J., Haldiman M., Bruschweiler B.J., Bochud M., Burnier M., Paccaud F., Dudler V. (2015). Cadmium body burden of the Swiss population. Food Addit. Contam. Part Anal. Chem. Control Expo. Risk Assess..

[51] De Craemer S., Croes K., van Larebeke N., De Henauw S., Schoeters G., Govarts E., Loots I., Nawrot T., Nelen V., Den Hond E. (2017). Metals, hormones and sexual maturation in Flemish adolescents in three cross-sectional studies (2002–2015). Environ. Int..

[52] Barr D.B., Wilder L.C., Caudill S.P., Gonzalez A.J., Needham L.L., Pirkle J.L. (2005). Urinary creatinine concentrations in the U.S. population: Implications for urinary biologic monitoring measurements. Environ. Health Perspect..

[53] Lin Y.S., Ho W.C., Caffrey J.L., Sonawane B. (2014). Low serum zinc is associated with elevated risk of cadmium nephrotoxicity. Environ. Res..

[54] Schnaper H.W. (2017). The tubulointerstitial pathophysiology of progressive kidney disease. Adv. Chronic Kidney Dis..

[55] Horiguchi H., Oguma E., Sasaki S., Okubo H., Murakami K., Miyamoto K., Hosoi Y., Murata K., Kayama F. (2013). Age-relevant renal effects of cadmium exposure through consumption of home-harvested rice in female Japanese farmers. Environ. Int..

[56] Oliver-Williams C., Howard A.G., Navas-Acien A., Howard B.V., Tellez-Plaza M., Franceschini N. (2018). Cadmium body burden, hypertension, and changes in blood pressure over time: Results from a prospective cohort study in American Indians. J. Am. Soc. Hypertens..

[57] Scinicariello F., Abadin H.G., Murray H.E. (2011). Association of low-level blood lead and blood pressure in NHANES 1999–2006. Environ. Res..

[58] Nakagawa H., Nishijo M. (1996). Environmental cadmium exposure, hypertension and cardiovascular risk. J. Cardiovasc. Risk.

[59] Kurihara I., Kobayashi E., Suwazono Y., Uetani M., Inaba T., Oishiz M., Kido T., Nakagawa H., Nogawa K. (2004). Association between exposure to cadmium and blood pressure in Japanese peoples. Arch. Environ. Health.

[60] Food and Agriculture Organization of the United Nations (FAO) World Health Organization (WHO) Summary and Conclusions In Proceedings of the Joint FAO/WHO Expert Committee on Food Additives Seventy-Third Meeting, Geneva, Switzerland, 8–17 June 2010. http://www.who.int/foodsafety/publications/chem/summary73.pdf.

