# Supplementary material for: A Comparison of the Nephrotoxicity of Low Doses of Cadmium and Lead
Source: Toxics. 2020 Mar 2;8(1):18. doi: 10.3390/toxics8010018 (PMC7151741; doi:10.3390/toxics8010018)
Supplement: Supplementary file 1 [file toxics-08-00018-s001.pdf]

Supplemental Material: A Comparison of the Nephrotoxicity of Low Doses of Cadmium and Lead

Soisungwan Satarug, Glenda C. Gobe, Pailin Ujjin and David A. Vesey

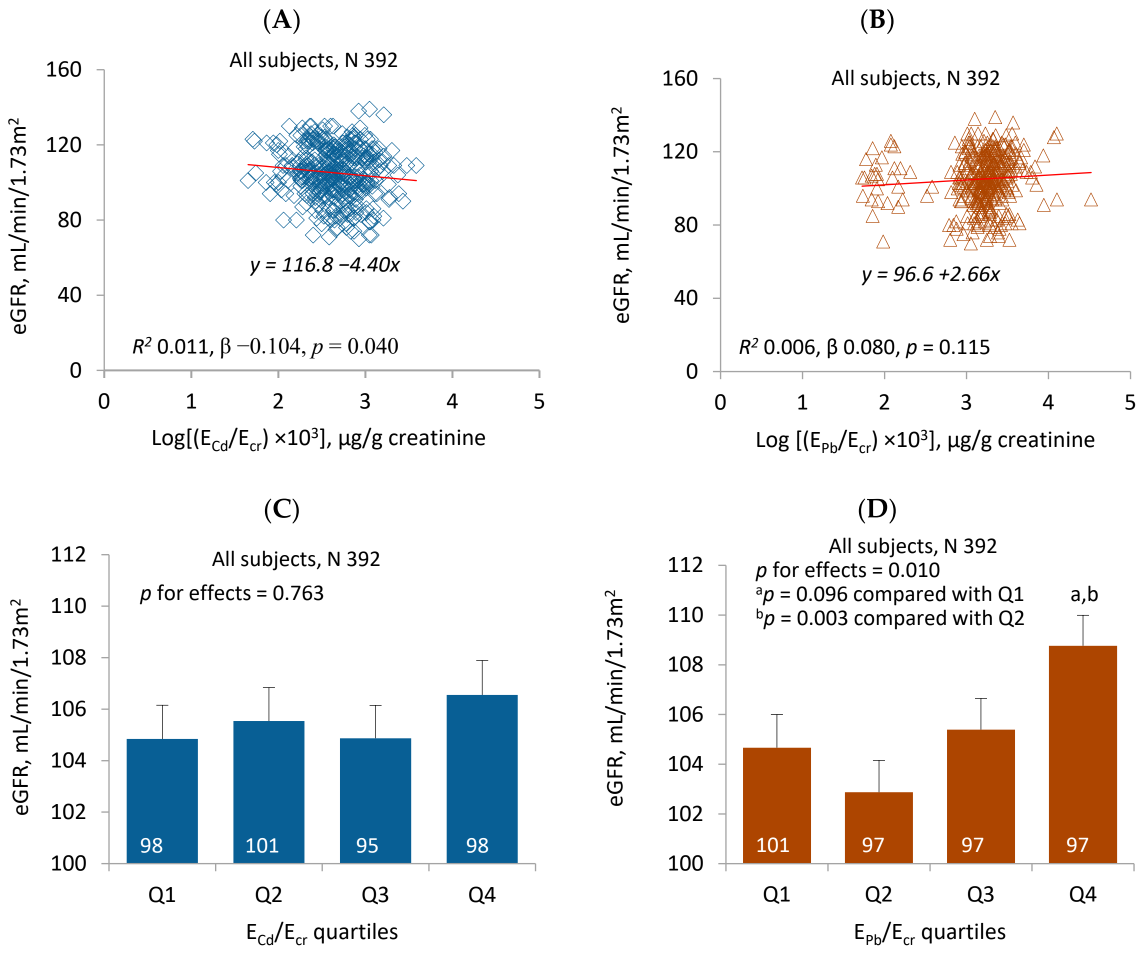

Figure S1. Comparing effects of E_{Cd}/E_{cr} and E_{Pb}/E_{cr} on eGFR change. The scatterplots relate eGFR to log [(ECd/Ecr) × 10^3] (**A**) and eGFR to log [(EPb/Ecr) × 10^3] (**B**) in all subjects. The linear equations and coefficients of determination (R^2) are provided together with standardized β and *p*-values. The bars represent the mean values for eGFR across E_{Cd}/E_{cr} quartiles (**C**) and E_{Pb}/E_{cr} quartiles (**D**) after adjustment for age, covariates and interactions. The numbers of subjects are provided for all subgroups. The geometric mean (GM) values (standard deviation) for E_{Cd}/E_{cr} in quartiles 1, 2, 3 and 4 are 0.14 (0.06), 0.35 (0.06), 0.58 (0.09) and 1.13 (0.51) μg/g creatinine, respectively. The GM (SD) for E_{Pb}/E_{cr} in quartiles 1, 2, 3 and 4 are 0.49 (0.43), 1.52 (0.14), 2.03 (0.19) and 3.52 (3.56) μg/g creatinine, respectively.

Table S1. Multivariable regression analysis for association of eGFR with E_{Cd}/E_{cr} and E_{Pb}/E_{cr}.

Independent Variables	eGFR, mL/min/1.73 m²									
	All, *n* = 392		Men, *n* = 195		Women, *n* = 197		Non-Smokers, *n* = 295		Smokers, *n* = 97	
	β	*p*	β	*p*	β	*p*	β	*p*	β	*p*
Age	−0.474	<0.001*	−0.564	<0.001*	−0.402	<0.001*	−0.445	<0.001*	−0.548	<0.001*
BUN	−0.144	0.002*	−0.103	0.100	−0.158	0.017*	−0.127	0.020*	−0.189	0.031*
E_{Cd}/E_{cr}	0.001	0.985	0.069	0.304	−0.043	0.516	0.016	0.779	−0.014	0.876
E_{Pb}/E_{cr}	0.044	0.365	0.015	0.806	0.065	0.324	0.043	0.460	0.012	0.888
Ferritin	0.067	0.216	0.141	0.024*	−0.013	0.838	0.048	0.429	0.098	0.246
Gender	0.181	0.008*	−	−	−	−	0.158	0.022	−	−
Smoking	0.039	0.481	0.024	0.696	−	−	−	−	−	−
Adjusted R^2	0.252	<0.001†	0.307	<0.001†	0.203	<0.001†	0.217	<0.001†	0.350	<0.001†

eGFR is a continuous dependent variable. Independent variables are listed in the first column, including E_{Cd}/E_{cr} as log $[(E_{Cd}/E_{cr}) \times 10^3]$, µg/g creatinine and E_{Pb}/E_{cr} as log $[(E_{Pb}/E_{cr}) \times 10^3]$, µg/g creatinine. A standardized regression coefficient β indicates the strength of an association between eGFR and an independent variable. *$p \le 0.05$ identify statistically significant associations. Adjusted R^2 value indicates the fraction of eGFR variation explained by independent variables. †$p \le 0.05$ indicate the model explained a significant variability of eGFR levels.

Table S2. Prevalence odds ratios for reduced eGFR across E_{Cd}/E_{cr} quartiles and E_{Pb}/E_{cr} quartiles.

Independent Variables/Factors	eGFR Levels <96 mL/min/1.73 m²				
	β Coefficients (SE)	POR [a]	95% CI Lower	95% CI Upper	*p* Value
Age (years)	−0.080 (0.015)	0.923	0.896	0.951	<0.001*
Gender	−0.685 (0.353)	0.504	0.252	1.007	0.052
Smoking	−0.170 (0.354)	0.843	0.421	1.690	0.631
Low body iron store status [b]	0.072 (0.426)	1.075	0.466	2.479	0.866
E_{Cd}/E_{cr}, µg/g creatinine					
Q1 (0.03–0.25)	Referent				
Q2 (0.26–0.44)	−0.125 (0.351)	0.883	0.444	1.755	0.722
Q3 (0.45–0.75)	0.059 (0.366)	1.061	0.517	2.176	0.872
Q4 (0.76–3.84)	0.357 (0.405)	1.430	0.646	3.162	0.378
E_{Pb}/E_{cr}, µg/g creatinine					
Q1 (0.05–1.24)	Referent				
Q2 (1.25–1.75)	−0.169 (0.380)	0.844	0.401	1.777	0.655
Q3 (1.76–2.41)	−0.689 (0.372)	0.502	0.242	1.042	0.064
Q4 (2.42–33.1)	−0.359 (0.395)	0.698	0.322	1.514	0.363

[a] POR = Prevalence Odds Ratios for eGFR levels ≤96 mL/min/1.73 m². The eGFR 96 mL/min/1.73 m² corresponds to the 25th percentile eGFR. [b] Low iron store status is defined as serum ferritin levels ≤ 30µg/L. *$p \le 0.05$ indicate a statistically significant increment of POR, compared with the reference. The GM (SD) for E_{Cd}/E_{cr} and E_{Pb}/E_{cr} together with number of subjects in all urinary Cd quartiles and urinary Pb quartiles are as in Figure S1.
